# Ovine Paratuberculosis Control in Australia Revisited

**DOI:** 10.3390/ani10091623

**Published:** 2020-09-10

**Authors:** Peter Windsor, Richard Whittington

**Affiliations:** Sydney School of Veterinary Science, The University of Sydney, Camden, NSW 2570, Australia; richard.whittington@sydney.edu.au

**Keywords:** Johne’s disease, sheep, Mycobacterium avium subspecies paratuberculosis, control

## Abstract

**Simple Summary:**

Ovine Johne’s Disease (OJD) is caused by *Mycobacterium avium* subspecies *paratuberculosis* (*MAP*) and is a less serious animal health issue in Australia than it was 10–20 years ago, with abattoir surveillance confirming declining prevalence. Control strategies for paratuberculosis potentially include (i) test and cull programs; (ii) management interventions to reduce faecal–oral transmission; and/or (iii) vaccination to limit and suppress infection, with the decline in OJD concern in Australia mostly attributable to vaccination programs providing effective disease suppression. However, as disease spread has continued, control program extension renewal to encourage the safe and wider use of vaccination, plus address misinformation promulgated by some disaffected producers, is required. As vaccination for OJD has contributed significantly to the welfare of Australian sheep, the livelihoods of producers, and reduced risk of *MAP* entering the human food chain, it should be more widely adopted globally.

**Abstract:**

OJD is no longer the serious animal health issue that it was for many Australian rural communities a decade and a half ago. Despite declining OJD prevalence as determined by abattoir surveillance, the disease continues to spread, with OJD extension programs required to continually address the misinformation promulgated by some disaffected producers as new areas have become affected. Improved regional and on-farm biosecurity, including the introduction of a risk-based trading system, may have contributed to improved attitudes to OJD control, although attitudinal differences between OJD endemic areas and where the disease is not well established remain. Declines in on-farm OJD prevalence are almost certainly attributable to the widespread uptake of vaccination programs, although encouraging the ongoing use of vaccination to prevent recrudescence and improved biosecurity when mortalities disappear, remains challenging. Vaccination has provided a robust strategy for managing OJD and contributed significantly to the health of Australian sheep and the lives of producers with affected properties. As vaccination offers a pathway to reduce the risk of *MAP* infection entering the human food chain from small ruminant products, it should be more widely adopted globally, accompanied by research efforts to improve efficacy and importantly, the safety of vaccination to both operators and livestock.

## 1. Introduction

Paratuberculosis or Johne’s Disease (JD), caused by several strains of *Mycobacterium avium* subsp. *paratuberculosis (MAP),* is an insidious, chronic disease of the global ruminant industries, causing mortalities and reduced production; interference in trading; and, in Australia, an extended period of profound negative social impact on rural communities involved in wool sheep production [1]. Johne’s disease was first described in cattle in 1895 as an intestinal condition that partially resembled tuberculosis. With increasing prevalence, geographic distribution and host range over the past 120 years, it has created serious concerns for animal health authorities due to the prolonged periods of incubation prior to clinical expression, plus faecal shedding of the causative bacterial agent *Mycobacterium avium* subsp. *paratuberculosis* (*MAP*) in faeces in subclinical phases. As diagnostic tests are of limited efficiency in the pre-clinical period, control of the disease is problematic due to difficulties in identification and the removal of infected animals [1].

Although bovine JD (BJD) has been recognized in Australia for about a century, Ovine Johne’s Disease (OJD) was not described in Australian sheep until the 1980’s [2]. OJD then slowly emerged until it was recognised by the turn of this century as the most serious of concerns for the sheep industry, particularly in NSW [3,4]. That OJD caused severe mortalities in many flocks in Australia is widely known, although initially there was considerable resistance to accepting that this was an industry-wide national issue, particularly until the range of economic losses was demonstrated [5,6]. These losses seriously compromised the financial stability of numerous wool sheep producers during a period when OJD caused depressed land values in southern NSW in the late 1990’s and early millennium, occasionally resulting in psychological stress and suicide risk [1].

Controversial food safety concerns have continued to “confound” research in relation to *MAP* and Crohn’s disease in humans. This is despite identification that people with Crohn’s disease had a seven-fold greater odds of *MAP* infection than non-Crohn’s sufferers, the increasing evidence of an association of Crohn’s disease and paratuberculosis, and acceptance by “experts” that *MAP* is a zoonosis [7,8]. Whilst a meta-analysis on Crohn’s and *MAP* concluded that there is an association, public health control measures beyond those provided by animal health control programs that reduce *MAP* in the food chain have been suggested as unjustified at present due to knowledge gaps in the role of *MAP* in human disease [9]. Nevertheless, some countries, including Norway, with a “stamping out” program, and Japan, with a program for removal of *MAP*-infected livestock from the food chain, have instituted stringent regulatory controls on paratuberculosis, and others have advocated for industry-led control programs for Johne’s disease. In a recent survey of 48 countries, paratuberculosis was confirmed to be very common in livestock, with more than 20% of herds and flocks infected with *MAP* in half of these countries, but formal control programs were present in only 22 countries [10]. Control programs were justified most commonly on animal health grounds, with protection of market access and public health also of relevance. Government funding was involved in about two thirds of countries, but operations tended to be funded by farmers and their organizations and not by government alone. The majority of countries (60%) had voluntary control programs. Generally, programs were supported by incentives for joining, financial compensation and/or penalties for non-participation, although security of funding for long-term control activities was a widespread problem. Control programs were reported to be successful in 16 (73%) of the 22 countries. Recommendations for future control programs include a primary goal of establishing an international code for paratuberculosis, leading to universal acknowledgment of the principles and methods of control in relation to endemic and transboundary disease. A holistic approach across all ruminant livestock industries is required for control of paratuberculosis because *MAP* is exchanged between species and there must be long-term commitment [10].

Control of paratuberculosis in sheep in Australia has largely depended on persistent use of an “old technology” vaccine (killed whole bacteria) (Gudair™, Zoetis, Australia) that appears to provoke generalized up-regulation of cell-mediated immunity and humoral immunity [11,12,13]. However, addressing concerns and knowledge gaps in the control of *MAP*, particularly in other species, requires improved understanding of the immunopathogenesis of paratuberculosis [9]. This may lead to the required improvements in both (i) the accuracy of diagnostic tests in identification of subclinical infectious so these animals can be removed before becoming infectious; and (ii) more efficacious vaccines that can prevent infection and faecal shedding, rather than depend on *MAP* suppression for their efficacy.

## 2. Pathogenesis of Paratuberculosis

The appearance of Johne’s disease in a sheep flock is a complex interplay between events at population, whole animal, tissue and cellular levels, as reviewed [14].

Population level events involve exposure to *MAP* with infection of a proportion of subjects at risk, with only some (5–15%, although rarely up to 25% in some flocks) developing clinical disease and dying. However, clinical cases are the “tip of the iceberg” due to high rates of subclinical infection. It has been shown that the presumed pathway from silent infection with no faecal shedding of *MAP*, through subclinical infection with light faecal shedding, to heavy faecal shedding and clinical disease, is incorrect. Surgical biopsies of infected animals examined over 3 years confirmed that a proportion of individuals avoided infection or resisted colonization by *MAP*, with some infected then recovering, whilst others progressed to clinical disease [15]. Understanding the pathways that determine these outcomes has progressed with work in an infection model in sheep [16,17,18]. Host dependency (age, genotype) and environment (stress, nutrition and dose of *MAP*) are considered factors of relevance, with Merinos and other breeds developing clinical disease, although some breeds may show earlier clinical presentation than others. This is accompanied by changes in early immunological pathways, including lymphocyte proliferation, apoptosis and cytokine activity, that are detected in blood cells of sheep following *MAP* exposure and lead to eventual disease expression in paratuberculosis [16,17,18].

Whole animal events involve colonisation of host tissues by *MAP*, and this occurs before inflammatory reactions are visible. The silent period of infection lasts for variable periods until granulomatous inflammatory foci arise that may become generalized, with shedding of *MAP.* There is a systemic cellular immune response that can be detected by the intradermal skin test, lymphocyte proliferation assay or whole blood IFN-gamma tests, indicating exposure to *MAP* and offering potential early detection of exposed individuals and farms, although they are still unable to distinguish between active infection and exposure. A switch from a cell mediated (Th1-like) response to a humoral antibody (Th2-like) response associated with progression of the infection to more severe forms has been proposed for paratuberculosis, although switches from Th1 to Th2 vary between sheep and are not observed at all in some animals [19,20].

Tissue events arise due to an interaction between *MAP* antigens and the antigen-specific CD4+ and CD8+ T cells in gut lymph nodes that are required for an effective immune response. Although B cells are activated to release specific antibody, this humoral response is ineffective as *MAP* is intracellular, although recently, gut antibodies have been suggested to have a role in protecting against recolonisation by *MAP* in sheep previously exposed to infection. It is the activated T cells returning to the intestine and interacting with infected phagocytes that produces the typical granulomatous inflammatory responses. Growing CD4+ cells and gamma-delta T cells are found in the Peyer’s patches in young sheep a few weeks after exposure to *MAP*, with granulomas eventually occupying the lamina propria. These enteric lesions appear to progress from paucibacillary to multibacillary, although mild paucibacillary lesions can regress [15]. Factors determining the switch from silent to active clinical disease are uncertain (although a novel regulatory pathway related to this disease switch has been identified in experimental sheep models), as are mechanisms enabling *MAP* to disseminate to other tissues, including within macrophages to the intestinal lumen, and then release to initiate faecal shedding.

Cellular events in *MAP* infection follow entry via epithelial cells, presumably involving numerous receptors on cell membranes of the intestinal mucosa. Infection occurs within 30 mins of *MAP* contact with M cells and goblet cells, with mycobacteria passed rapidly to macrophages to which they attach and enter, mediated by the fibronectin receptor and components of the *MAP* cell wall. The host responds when pathogen-associated molecular patterns (PAMP) such as cell wall lipoproteins engage the host’s pathogen recognition receptors (PRR) such as the toll-like receptors (TLR), leading to the induction of cytokines and activation of innate immune mechanisms. TLR2/TLR6 heterodimers are involved in the recognition of mycobacterial antigens. The fact that pathogenic mycobacteria survive phagocytosis, acquire nutrients for growth within the phagolysosome, spread between macrophages, disseminate within them and finally escape the confines of the macrophage to infect additional animals is fundamental to *MAP* pathogenesis. Mycobacteria are passed from infected macrophages to uninfected macrophages, presumably by apoptosis, and *MAP* alters its gene expression within macrophages, using numerous metabolic pathways and dozens of altered proteins to withstand stresses such as hypoxia, nitrosative and oxidative conditions, and nutrient depletion compared to optimal growth conditions. Gene expression studies in paratuberculosis have identified that *MAP* may utilise lipids as an energy source once within the macrophage, with several gene families differentially regulated, including S100 calcium binding, lysozyme function, MHC class I and class II, T cell receptor and transcription factors [21]. These differentially regulated genes are considered as putative biomarkers of *MAP* exposure, or of the specified disease or resilience outcomes.

## 3. Ovine Paratuberculosis Control Strategies

Control strategies for paratuberculosis include the following:

1. Test and cull programs;
2. Management interventions to reduce faecal–oral transmission;
3. Vaccination to limit and suppress infection [1].

In extensive sheep flocks in Australia, it is the latter strategy that has proven to be most successful. However, further research solutions may provide more effective JD control programs, a priority being the development of tests that can detect late subclinical infections to enable removal of future sources of infectious material from flocks/herds and the food chain, plus predict the likely outcomes of animals exposed to the organism at an early age. The recent developments in OJD control include an improved understanding of the immune and cellular profiles of sheep with varying paratuberculosis outcomes (as above), offering potential improved testing regimes, plus increasing recognition of the importance of ongoing annual lamb vaccination and improved biosecurity to reduce losses and risks of disease [1,4]. However, improving national paratuberculosis control programs should also be a priority to manage disease risk from trade. Strong leadership and communication from livestock health authorities is required to build trust within rural communities confused by the difficulties in managing this insidious disease.

Change management considerations provide a framework for reflection on animal health strategies, potentially enhancing our understanding of the strengths and weaknesses of national programs that are driven by livestock industry stakeholders keen to enhance the welfare of animals and quality of ruminant-derived products [1,4]. However, control of paratuberculosis in Australia by government regulation, de-stocking and other approaches generally failed to prevent or control the disease or build the trust required for producer compliance with these approaches [1,4]. The OJD situation in NSW in particular, the state with the largest sheep numbers where the disease was first established and recognized, precipitated into a rural community crisis until the introduction of Gudair™ vaccination in 2002. Vaccination was then supplemented by the introduction of the “sheep health statement”, a vendor declaration system to facilitate disease risk awareness during the trading of sheep, plus eventually a more effective program of general biosecurity risk awareness, supported by legislation.

## 4. Misinformation on OJD: The “Top 10” List

The history of OJD in Australia should acknowledge the considerable frustration periodically expressed that surrounded this prolonged animal health program. In particular, the emergence of misinformation, increasingly facilitated by the rise of social media, created confusion and, occasionally, media-facilitated attacks on the “messengers” (including researchers) when emerging scientific information did not fit well with pre-conceived views on OJD control issues. Revisiting this misinformation issue may be of interest to the “survivors” from this difficult period of rural community turmoil. On reflection, it may also have been an early warning of what was to come from emerging global scientific “denialism” and politicization of science. Sharing this experience is offered as a warning to others, particularly those more recently challenged by OJD incursions and those that may or soon will be considering *MAP* disease control programs.

1. That the Australian climate is not conducive to OJD spread and OJD has always been present were common claims in the intervening years between the first diagnosis of OJD and the recognition that it was becoming a serious epidemic. Studies on survival of *MAP* in soil plots conclude that different ecosystems affect *MAP* survival rates, but the disease has generally emerged in most areas that have received numbers of infected sheep [1,2,3,10]. That the disease was always present was promulgated by several industry leaders who claimed that as “skinny sheep” have always occurred on Australian farms, even when drought was absent, and the animal health surveillance systems prior to 1980 were too incompetent to have detected OJD. This scenario is considered very unlikely considering this was the “golden age” of diagnostic pathology when many regional veterinary laboratories were established with highly trained pathologists, funded partially through the substantial program that eliminated bovine tuberculosis and brucellosis in Australia in the 1970–80’s.

2. That OJD is due to mismanagement and of no economic importance was a common claim by those who were yet to recognize the disease (often despite that it was occurring on neighboring farms), or were fortunate to live in an area where the disease did not appear to be readily established (e.g., New England, NSW). Anecdotal reports that the most severe outbreaks occurred on “cell-grazing” farms, and that mortalities persisted in some vaccinated flocks that grazed ewes in restricted “river flat” areas during drought, is consistent with intensive grazing being a risk factor for high prevalence of OJD. The cost of the disease and the benefits of vaccination are now well established in Australia [1,5,6,11].

3. That OJD strains are species-specific and OJD risk is breed-specific and only affects older animals were widely held beliefs in earlier years of OJD control, with the “S” strains fastidious in culture and appearing to be confined to sheep (or fiber goats in contact with sheep). These claims are now considered erroneous. Ovine strains infect other species and breeds (although Merinos are more susceptible) despite differences in disease susceptibility involving variations in immunological mechanisms between and within species and breeds [22]. Age factors are of interest, as vaccination trials show that weaner sheep can shed *MAP* and may die as hoggets, with younger animals more likely to be affected as the within-flock prevalence rises [23].

4. That OJD expression is almost entirely related to soil factors, particularly pH and Iron, was promulgated by a persistent group of commentators that failed to understand the complex web of host–pathogen–environment interactions in disease pathogenesis. This diverted research funds towards proving an hypothesis that OJD was “all about the soil”, with nil results, other than the useful risk factor study that failed to support the soil hypothesis [24].

5. That vaccination adverse effects are too severe in humans and sheep for Gudair™ to be used safely, was an argument that delayed the introduction of vaccination to Australian farms where losses were unsustainable. Human safety concerns were correct, with several incidents of self-injection demonstrating that the standard of vaccine administration by farmers was poor, although this risk has now been well managed since the introduction of the Sekurus^®^ injecting device [1,25,26,27].

6. That vaccination lesions are breed dependent has had some support from field observations (e.g., Suffolk sheep are considered by some to be more likely to develop large granulomas). However, there are many variables that could impact on these field observations and the severity of necrosis that is induced by maladministration of Gudair™ vaccine to both sheep and humans, particularly when injected into muscle, is generally insufficiently understood by producers [1,12].

7. Vaccination for 3 years removes OJD-attributable mortality, and vaccinates are safe to trade as persistent shedding in vaccinates simply reflects inadequate administration of vaccine. The emerging research that confirmed that shedding of *MAP* persisted in flocks vaccinating for extended periods was, unfortunately, derided by some producers, particularly studs keen to just “vaccinate and forget” and trade freely into areas where the disease was possibly absent [1,12,13].

8. That testing regimes are useless and a waste of time and money, reflects the difficulties of communicating the limitations in sensitivity and specificity of current testing methods for such an insidious disease, particularly with tests unable to detect early subclinical infections. Understanding of the concept that the PFC (pooled faecal culture test of 7 pools each containing a faceal pellet from 50 animals) has “a 95% confidence of detecting infection if prevalence is at 2% or greater” is limited, with farmers expecting results as either positive or negative, rather than expressed as probabilities. The issue of inability to detect early subclinical infection remains with q-PCR (real time polymerase chain reaction) and explains why a series of tests has been advocated [1,12,21,28].

10. OJD will spread throughout the entire Australian sheep industry, so everyone should just vaccinate and whole flocks should be vaccinated. This claim reflects the desire for open trade and frustration at state or regional controls on movement of sheep from infected areas. The history of OJD in Australia, despite its continuing spread, suggests that many areas of the country will likely remain either OJD-free or have a very low prevalence of OJD, and lesions detected at slaughter have commenced to decline [1,4]. Whole flock vaccination has occurred in a number of flocks, although the evidence from limited studies for efficacy of Gudair™ when vaccinating adult animals remains uncertain, at least in high-prevalence flocks [1,12,13].

11. That paratuberculosis as a zoonosis is a “beat-up” by veterinarians attempting to safeguard their jobs, was advanced by those in denial of the growing evidence that *MAP* or even *MAP DNA* exposure is likely to be associated with human Crohn’s Disease and possibly other bowel ailments. Some scientists now believe that *MAP* antigens can trigger aberrant inflammatory response in susceptible people, so whilst the aetiology of Crohn’s disease is clearly multifactorial, genetically determined susceptibility and exposure to triggering antigens appear to be two of the factors involved [1,7,8].

## 5. Conclusions

Despite paratuberculosis knowledge gaps remaining, OJD is no longer the serious agripolitical animal health issue that it was for many Australian rural communities a decade and a half ago. However, the disease continues to spread, with both OJD extension and funded research programs required to address the challenging issues of promulgated misinformation, and the need for continual improvement of diagnostic and disease control tools, respectively. Improved regional and on-farm biosecurity, including the introduction of a risk-based trading system, may have contributed to changing attitudes to OJD control, although decreasing on-farm OJD prevalence is almost certainly attributable mostly to the uptake of vaccination programs. Encouraging the ongoing use of vaccination and improved biosecurity when OJD mortalities disappear remains challenging. Vaccination has provided a robust strategy for managing OJD and contributed significantly to the health of Australian sheep and the lives of producers with affected properties. As vaccination offers a pathway to reduce the risk of *MAP* infection entering the human food chain from ruminant products, it should be more widely adopted globally.
